# Herbalists and the Panels

**Published:** 1913-05-03

**Authors:** Gordon P. Ward

**Affiliations:** *pro* General Secretary. National Medical Guild, 34 Villiers Street, Strand, W.C.


### Herbalists and the Panels.

*To the Editor of* The Hospital.

Sir,—In your issue before last you kindly printed a letter from the National Medical Guild relating to the employment of herbalists by Insurance Committees. Your leading article* implies that someone has suggested that herbalists are being admitted to the panel in some districts.

May we point out that the information on which our letter was based was only to the effect that herbalists were being recognised as persons with whom arrangements could be made outside the panel? This we were informed by an official was the case, and we are not aware that it has been in any way disputed. The matter seems to us sufficiently serious to warrant our calling your further attention to it.—I am, Sir, yours faithfully,

Gordon P. Ward, *pro* General Secretary.

National Medical Guild,
34 Villiers Street, Strand, W.C.,
April 22, 1913.

* A Note in our issue of April 19.

